# Self-navigated three-dimensional cardiac T_2_ mapping at 3T

**DOI:** 10.1186/1532-429X-15-S1-P51

**Published:** 2013-01-30

**Authors:** Ruud B van Heeswijk, Davide Piccini, Hélène Feliciano, Juerg Schwitter, Matthias Stuber

**Affiliations:** 1 Department of Radiology, University Hospital (CHUV) and University (UNIL) of Lausanne, Lausanne, Switzerland; 2 Center for Biomedical Imaging (CIBM), Lausanne, Switzerland; 3 Siemens Schweiz AG, Healthcare Sector, Lausanne, Switzerland; 4 Cardiology and Cardiac MR Center, University Hospital of Lausanne (CHUV), Lausanne, Switzerland

## Background

Cardiac T_2_ mapping using a variable T_2_ preparation module (T_2_Prep) has recently gained attention for its ability to quantify the extent of edema (Giri, JCMR 2009). Due to time constraints, the T_2_ maps are commonly acquired as one or several two-dimensional slices, while the underlying pathology has a three-dimensional (3D) structure. The next logical step would therefore be to exploit recent hardware and software advances to directly acquire 3D T_2_ maps. To this end, we tested the feasibility of using a self-navigated 3D radial acquisition with a variable T_2_Prep for 3D T_2_ mapping at 3T.

## Methods

Approval was obtained from the institutional review board. A 3D self-navigated undersampled balanced steady-state free precession (bSSFP) sequence (TR/TE=2.6/1.33ms, matrix 128^3^, flip angle 70°) with a spiral phyllotaxis radial 3D trajectory (Piccini, MRM 2011) was implemented on a 3T clinical system (Skyra, Siemens AG). This self-navigated pulse sequence allows free breathing acquisitions with 100% scan efficiency, while ECG triggering every 2 heartbeats and TE_T2Prep_=60/30/0ms allow for a total acquisition time of ~18min with an isotropic spatial resolution of (1.7mm)^3^. The datasets were registered using 3D affine registration (Studholme, Med Image Anal 1996). Through Bloch equation simulations, the heart-rate-dependent T_1_ -relaxation-related offset in the T_2_-fitting equation was ascertained. Subsequently, the validity and accuracy of the T_2_ fitting was tested in a phantom whose "true" T_2_ values were previously determined. The in vivo robustness of the T_2_ determination was then tested in 9 healthy adult subjects. Finally, the sequence was applied for the detection of edema in a 75-year-old male infarct patient after revascularization of his proximal left circumflex.

## Results

**Figure 1:**
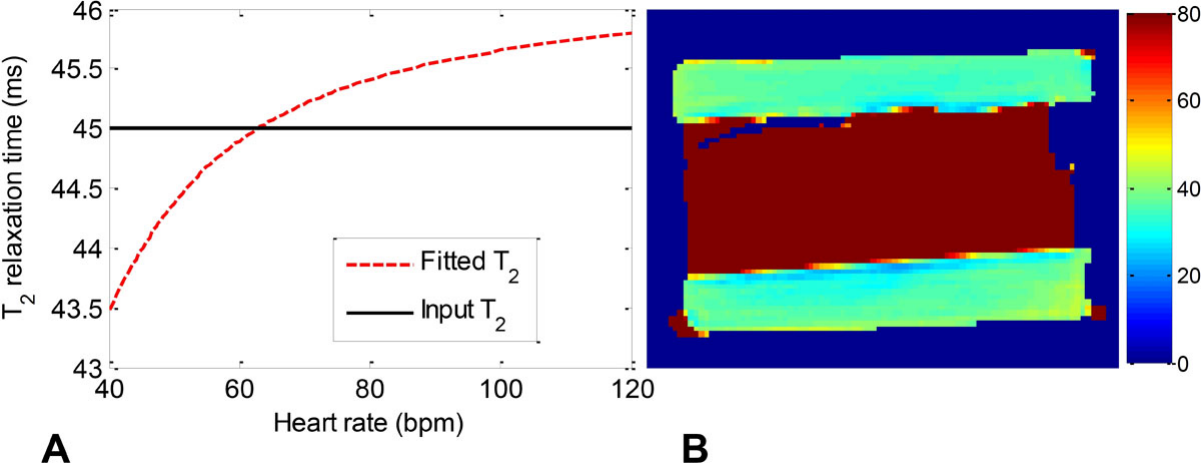
A) Bloch equation simulation demonstrating that the dependence of the T_2_ fit of the magnetization on the heart rate due to varying T_1_ relaxation is relatively low (between 43 and 46ms, a variation of 3%), while the "true" input T_2_ was 45ms. B) T_2_ map of a phantom that approximates arterial blood and myocardium. The T_2_ values of the two ‘myocardium' compartments (turquoise) are very similar at 35.3±2.1ms and 35.5±2.4ms and within 1% of the "‘true" T_2_ value of 35.6ms.

**Figure 2:**
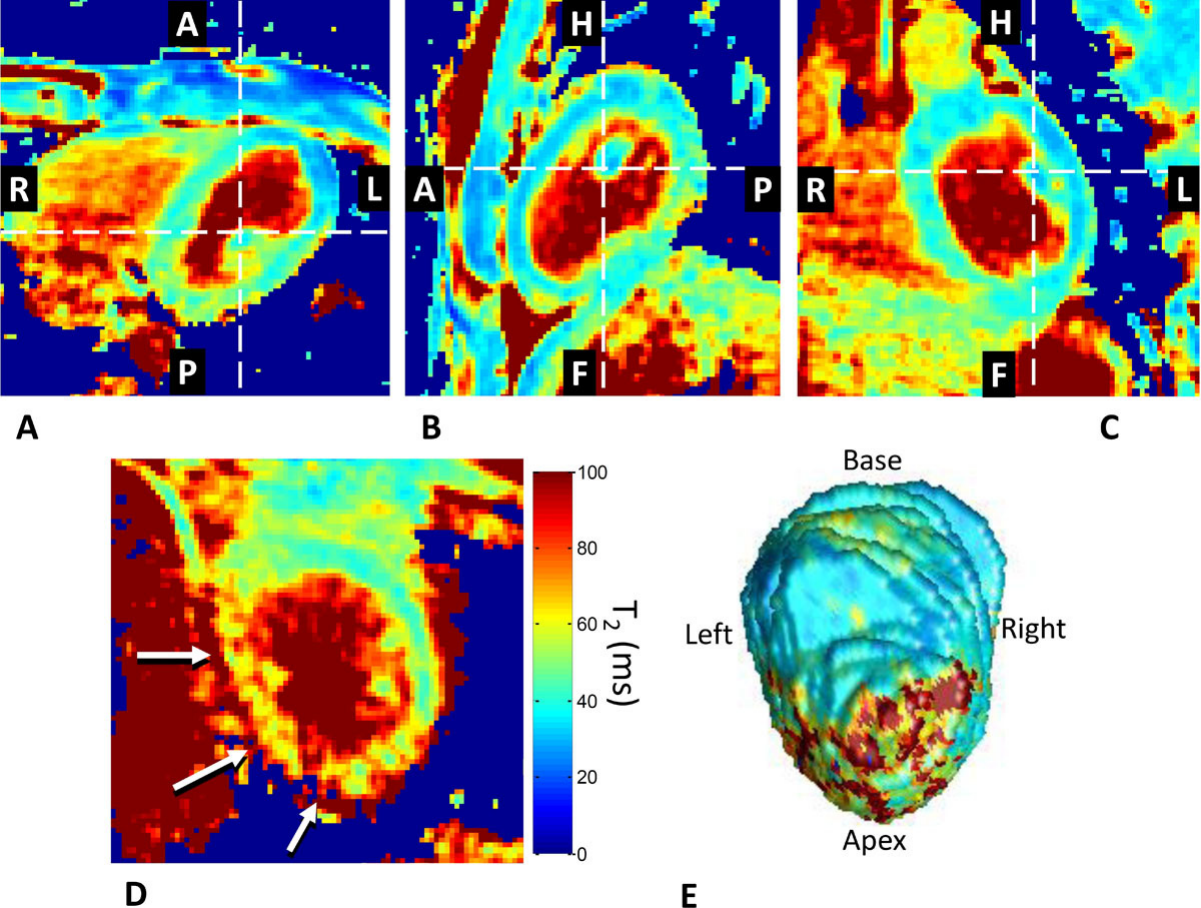
A-C) Axial, sagittal and coronal multi-planar reformatted T_2_ maps through the LV of a healthy volunteer. The myocardium is well defined and T_2_=41.3±2.1ms. D) A sagittal T_2_ map of a patient with a subacute myocardial infarction demonstrates elevated T_2_=62.4±9.2ms in the inferior and infero-lateral segments (arrows). E) 3D segmented LV at a sub-endocardial surface as seen from a posterior position, with a clearly visible inferior and infero-lateral infarction.

The Bloch equation simulations of the pulse sequence demonstrated that the input T_2_ value could be accurately fitted from the magnetization M with the equation [M=M _0_e ^-TET2Prep/T2^ +0.08M_0_], while the fitted T_2_ had only a ~3% variation over the common range of heart rates (Fig.1A). The phantom T_2_ maps demonstrated high homogeneity and fitting accuracy with the 3D sequence matching the ‘true' value to within 1% (Fig.1B). The volunteer study (Fig.2A-C) suggested good agreement with previously reported T_2_ values at T_2_=39.3±3.9ms (Van Heeswijk, JACC Imaging 2012, in press). A region of significantly elevated T_2_ (60.4±9.1 vs. 41.0±4.5ms) was identified in the patient in the infero-lateral myocardium of the left ventricle (Fig.2D,E), consistent with the findings on X-ray coronary angiography.

## Conclusions

The proposed technique provides an easy and time-efficient way to obtain accurate isotropic T_2_ maps of the whole heart. Accurate T_2_ values were obtained in the phantom, while those in volunteers are consistent with previously reported values. The preliminary patient study demonstrated elevated T_2_ in the infarcted region as expected.

## Funding

Foundation Emma Muschamp

